# Embedding Technology-Assisted Parenting Interventions in Real-World Settings to Empower Parents of Children With Adverse Childhood Experiences: Co-Design Study

**DOI:** 10.2196/55639

**Published:** 2024-11-22

**Authors:** Grace Aldridge, Ling Wu, Joshua Paolo Seguin, Jennifer Robinson, Elizabeth Battaglia, Patrick Olivier, Marie B H Yap

**Affiliations:** 1 Turner Institute for Brain and Mental Health School of Psychological Sciences Monash University Clayton Australia; 2 Department of Human Centred Computing Monash University Clayton Australia; 3 Family Services IPC Health Sunshine Australia

**Keywords:** co-design, service design, intervention, digital technology, parenting, children, technology, parenting program, health care services, adverse childhood experience, ACE, mental disorder, innovate, social services, community health, evidence-based, parenting intervention

## Abstract

**Background:**

Adverse childhood experiences are strongly associated with mental disorders in young people. Parenting interventions are available through community health settings and can intervene with adverse childhood experiences that are within a parent’s capacity to modify. Technology can minimize common barriers associated with engaging in face-to-face parenting interventions. However, families experiencing adversity face unique barriers to engaging with technology-assisted parenting interventions. Formative research using co-design methodology to provide a deep contextual understanding of these barriers can help overcome unique barriers and ensure these families can capitalize on the benefits of technology-assisted parenting interventions.

**Objective:**

This study aims to innovate the parenting support delivered by a community health and social service with technology by adapting an existing, evidence-based, technology-assisted parenting intervention.

**Methods:**

Staff (n=3) participated in dialogues (n=2) and co-design workshops (n=8) exploring needs and preferences for a technology-assisted parenting intervention and iteratively developing a prototype intervention (Parenting Resilient Kids [PaRK]-Lite). Parents (n=3) received PaRK-Lite and participated in qualitative interviews to provide feedback on their experience and PaRK-Lite’s design.

**Results:**

PaRK-Lite’s hybrid design leverages simple and familiar modes of technology (podcasts) to deliver intervention content and embeds reflective practice into service provision (microcoaching) to enhance parents’ empowerment and reduce service dependency. A training session, manuals, session plans, and templates were also developed to support the delivery of microcoaching. Feedback data from parents overall indicated that PaRK-Lite met their needs, suggesting that service providers can play a key role in the early phases of service innovation for parents.

**Conclusions:**

The co-designed technology-assisted parenting intervention aims to offer both parents and clinicians a novel and engaging resource for intervening with maladaptive parenting, contributing to efforts to respond to childhood adversity and improve child mental health. Future research in the field of human-computer interaction and health service design can consider our findings in creating engaging interventions that have a positive impact on the well-being of children and families.

## Introduction

### Background

The incidence of mental disorders in young people is strongly associated with adverse childhood experiences (ACEs) [1,2]. Unlike many community- or society-level ACEs, maladaptive parenting is an ACE that is potentially within a parent’s capacity to modify. Evidence-based parenting interventions designed to intervene with maladaptive parenting hold clear potential for preventing or reducing the impact of such ACEs and protecting young people from the risk of mental disorders. However, barriers to engaging in parenting interventions and services are especially prevalent for families who experience adversity, marginalization, and stress due to socioeconomic disadvantage [3,4]. Technology has the potential to minimize or overcome common barriers associated with engaging in face-to-face parenting interventions, such as time constraints and competing demands, and can hence help expand reach and improve the cost-effectiveness of parenting interventions [5]. Given intervention engagement is a key mechanism for improving target behavior [6,7], and features of technology-assisted parenting interventions can positively impact parental engagement [8], understanding factors that influence intervention engagement through formative research is critical to ensure families experiencing adversity can capitalize on the benefits of technology-assisted parenting interventions.

Technology-assisted parenting interventions can improve parenting outcomes (including maladaptive parenting behaviors) and reduce child internalizing problems [9,10], externalizing problems [11-13], and promote child physical and mental health [14]. Importantly, these interventions have also been found to be efficacious for families experiencing adversities such as socioeconomic disadvantage [15]. Technology-assisted interventions are also easier to scale compared to face-to-face interventions, enhancing cost effectiveness, long-term feasibility, and importantly reaching families experiencing socioeconomic stress or disadvantage [16]. Evidence-based parenting interventions designed to reduce the occurrence of ACEs or reduce their impact on child mental health are offered face-to-face in community settings in Australia [17]. However, mainstream uptake of technology to deliver child mental health care has historically been limited due to concerns relating to authenticity, privacy, security, access, and risk [18]. Furthermore, parents experiencing socioeconomic disadvantage can face unique barriers to engaging with technology-assisted interventions, such as lower digital literacy [19] and more limited access to devices [20], which can result in parents lacking confidence in navigating digital resources [21]. Embracing technology-assisted parenting interventions in these settings to capitalize on their benefits thus requires a deep contextual understanding to ensure program designs account for such needs and constraints.

Another barrier to parents’ engagement with innovative interventions can be the intervention practitioners’ low motivation or stress around delivering them, due to implementation difficulties [22,23]. Practitioners are more likely to adopt an innovative intervention when its design accounts for their needs and perspectives on its delivery, reflects a shared understanding of its meaning and value, and involves fewer changes to existing practices [24]. Practitioner skills and attitudes toward technology-assisted modalities used during COVID-19 were vital to the successful implementation of any service provided [25], consistent with past research linking acceptance of technology with users’ perceived competence and sense of achievement while using it [26]. Thus, involving practitioners in the design of an innovation is critical for increasing the likelihood of them adopting innovative, technology-assisted parenting interventions and, in turn, improving parents’ engagement with these interventions.

Innovating evidence-based interventions for different service contexts inevitably leads to differences from an intervention’s original design. To ensure innovations to evidence-based interventions can still be monitored for fidelity and rigorously evaluated, Greenhalgh et al [24] suggest embedding “hard cores” (nonnegotiable components such as evidence-based content) in “soft peripheries” (contextual structures and systems required for full implementation, such as human resources and technology platforms for delivery). “Soft peripheries” may thus represent the design space for innovation. A common challenge in co-designing innovations is the additional time required to iteratively explore and test concepts [27]. Methods that minimize the burden on co-designers, such as involving them in designing certain points on the client journey rather than the whole journey [28], thus warrant further exploration.

Practitioners hold specialized knowledge about their service’s contextual structures and systems that can feasibly be adapted; hence, they are well placed to first determine appropriate intervention features and co-develop innovations. Practitioners who support and advocate for the needs of families whose children experience adversity also possess insights into the lived experience of these families, which can be leveraged during this process. It is critical to involve intervention users in a design process; however, for families experiencing socioeconomic disadvantage and stress, such involvement may place further pressure on their already-limited resources. This can be overcome by delivering prototype innovations to these families as part of an iterative co-design method to obtain experience-based feedback to understand which features are most accessible and engaging [21], while simultaneously offering families benefits through accessing additional support to what they might usually receive. This feedback can inform future, larger-scale iterations until an intervention is sufficiently refined to be implemented at scale.

Parenting Resilient Kids (PaRK) is a web-based preventive parenting intervention designed to equip parents with evidence-based parenting strategies to reduce their child’s risk of depression or anxiety problems [29-31]. PaRK has been shown to improve self-reported parenting behaviors, including maladaptive parenting behaviors [2,32,33]. These improvements were also significantly associated with parental engagement with the intervention [34], supporting intervention engagement as a key mechanism for target behavior change [7]. Thus, PaRK has the potential to complement intervention pathways in community service organizations offering evidence-based parenting interventions designed to reduce the occurrence of ACEs and help improve outcomes for families accessing them.

PaRK’s “hard cores” are the evidence-based parenting guidelines [35] that underpin the key messages in the program content (presented in its original format through a self-assessment tool, individualized feedback, and module content [29]). Prior research with parents experiencing socioeconomic disadvantage has suggested simple, easy-to-understand language, easy-to-navigate user interfaces, and flexible intervention navigation as intervention features that would enhance their engagement in technology-assisted parenting interventions [21]. Such features represent the “soft peripheries” of PaRK that should be explored with service providers and parents whose children experience adversity, to inform how they may best be adapted or redesigned to meet their needs.

### This Study

The aim of this study was to innovate a community health and social service’s parenting support with technology by adapting PaRK. To do this, this study first used co-design methods to (1) understand the needs and preferences of service provider practitioners (who provide support to parents of children who have experienced ACEs, hereon referred to as “service providers”) when it comes to delivering a technology-assisted parenting intervention (PaRK) in their service context and (2) design and develop a prototype parenting intervention (using PaRK) for the health service. This study then aimed to (3) deliver the prototype intervention to parents and (4) understand how its design met parents’ needs and preferences for the adoption of PaRK and if further adaptations are needed.

## Methods

### Ethical Considerations

The study protocol and procedures were approved by the Monash University Human Research Ethics Committee (28222). Each participant was provided with an explanatory statement and the opportunity to ask questions about their involvement before providing consent to participate. Participants were able to opt out at any time. All reported data were anonymized and identifiable raw data were stored on a secure server on a password-protected computer at Monash University. Only Monash University researchers named on the approved ethical protocol had access to these data. Service provider participants were not financially compensated as all research activities took place during their scheduled hours. Parents who consented and participated were reimbursed Aus $35 (US $23.6) for each hour spent engaging with the intervention and feedback interview.

### Study Setting

This study was affiliated with a larger research intervention conducted by the Centre for Research Excellence in Childhood Adversity and Mental Health. It was conducted in the City of Brimbank, a local government area of Greater Metropolitan Melbourne, Victoria, Australia, which is culturally diverse and experiences greater socioeconomic disadvantage and a substantially higher proportion of developmentally vulnerable children compared with Greater Metropolitan Melbourne [36]. We partnered with a large, multisite provider of community health services within the City of Brimbank and other culturally diverse local government areas with higher concentrations of socioeconomic disadvantage. This community health service provides Family Services, a free, state-wide, first port-of-call service for families who have experienced or are at risk of experiencing adversity or becoming involved with Child Protection (Victoria’s state-wide service for children and young people who have experienced significant harm within the family). Family Services comprises 2 service elements: *medium-term casework*, in which a family receives a comprehensive needs and risk assessment and multidisciplinary intervention responses (such as therapeutic home-based interventions, advocacy, crisis intervention, and counseling) and *active holding responses*, in which a family receives low-level monitoring and support until allocated to a caseworker, or short-term intervention that could lead to case closure. Given this study’s focus on promoting parenting interventions to prevent or reduce the risk of ACEs, the Family Services intervention was identified as an appropriate design space in the community health service.

### Recruitment

#### Overview

This study and its primary outcome are the first iterations of a larger design process guided by the Double Diamond model [37]. Designing, developing, and piloting a minimum viable product is considered a cost- and time-efficient way of designing scalable and sustainable health services, especially in low-income regions [38]. A small sample size is considered appropriate for a small-scale qualitative pilot study, such as the study by Vasileiou et al [39], especially when in-depth exploration that involves deep, case-oriented analysis is used as the primary method for design inquiry [40]. We therefore engaged a small number of service providers to gain an in-depth understanding of their service provision at this initial stage and strategically engaged parents at times of necessity. Results from this small-scale study will support and guide future, larger-scale iterations until the intervention is sufficiently refined to be implemented at scale.

#### Service Providers

Service provider recruitment occurred from June to July 2021. A total of 28 eligible service providers were identified by the Family Services manager, who acted as the link between researchers and community health service clinicians throughout the study. An email, including a web-based flyer, was sent to eligible staff by the Family Services manager on behalf of the researchers. Any interested staff were instructed in the email to contact the first author directly to express their interest and provide consent. The first author organized brief meetings with interested staff via Zoom (Zoom Video Communications) to provide further information and address any questions about the research. All research activities took place during service providers’ scheduled hours, meaning their involvement was logged as intervention development.

#### Parents

Parent recruitment occurred from May to June 2023. In total, 7 parents living in Wyndham with children aged between 4 and 11 years were invited to receive the co-designed intervention and then participate in an interview to provide feedback on their experience. Parents were recruited via two methods. In the first method, Family Services practitioners working with parents of children aged between 4 and 11 were sent a study flyer for parents (in soft and hard copy form), and they were invited to pass the flyer on to parents who were eligible and potentially interested in receiving parenting support during their usual service contact with parents. In the second method, parents who have been involved in prior research projects occurring at the community health service and who indicated an interest in future research projects occurring as part of the Centre for Research Excellence in Childhood Adversity and Mental Health were emailed a study flyer by the first author, which included an invitation to directly email the first author to express their interest. The first author then organized a brief telephone call with interested parents to provide them with an opportunity to ask questions about the study before deciding and consenting to participate.

### Study Design

The co-design method for this study was guided by the Double Diamond design model [37]. The first diamond’s aim is to *discover* and *define* the design problem, and the second diamond’s aim is to *develop* and *deliver* possible solutions.

Phase 1 of this study involved dialogues and co-design workshops to *discover* and *define* design problems and the design space for a technology-assisted parenting intervention with service providers. This phase was conducted while Melbourne’s 2021 COVID-19 lockdown restrictions were in place. As these restrictions placed additional stress on service providers who were in the process of pivoting to technology and working from home while supporting vulnerable families, we selected co-design methods that placed minimal time and cognitive burden on service providers while providing the researchers with substantial experience-based insights to leverage the design process.

Phase 2 involved co-design workshops to *develop* prototype solutions with service providers. Phase 3 involved piloting the prototype to *deliver* it to parents and conducting qualitative feedback interviews to inform the next iteration of development, reflecting the framework’s core principle of iteration. A summary of co-design methods and aims can be found in Multimedia Appendix 1. The remainder of this section reports methodological details and results of co-design activities in each phase.

## Results

### Phase 1: Identify Design Problem and Space

#### Dialogues

##### Overview

Service provider participants (n=2) were aged ≥18 years and held tertiary-level social work qualifications. One service provider provided *medium-term casework*, while the other provided *active holding responses*; hence, between them, the 2 service elements in Family Services were covered. Both dialogues were conducted in individual formats during July 2021. One was conducted face-to-face (on the day before lockdown restrictions were introduced in Melbourne, Victoria), and the other was conducted via Zoom as COVID-19 restrictions prevented face-to-face meetings. Dialogues lasted for 2 hours (including a 10-minute break) and followed an unstructured format to enable free flow of conversation. “Horizontal” questions were used by the researcher to understand the range of service providers’ experiences (ie who, what, where, and how), and “vertical” questions were used to uncover root causes or deeper beliefs underpinning service providers’ experiences or ideas about using technology. The first author also used verbal and visual methods to take notes, where visual methods, such as sketching, prompted deeper thinking about conceptual relationships [41]. A relational approach to communication was fostered as service providers’ knowledge and identity became associated with the researcher’s empathy and active listening.

Dialogue recordings were transcribed verbatim by an automatic artificial intelligence transcription service (Descript) and cleaned by the first author. Inductive content analysis was used for its flexible approach to analyzing text data [42]. Data were coded according to an “empathy map” [43], that is, what service providers “do, say, think, and feel” for the researcher to empathically understand service providers’ experience. Common themes between service providers were then openly coded and grouped into higher order key design considerations following the approach to inductive content analysis outlined by Elo and Kyngäs [44].

##### Dialogue Theme 1: Parent Vulnerability Interferes With Confidence and Motivation to Engage

Participants discussed parents’ vulnerability upon entering Family Services due to the fear that child protection could become involved or fear of stigma from needing help and additional stressors, such as mental health problems, family violence, and financial stressors (many of which were exacerbated by COVID-19 lockdowns and requirements to provide homeschooling to children), interfering with their confidence and motivation to keep trying new parenting strategies. They wondered whether this vulnerability might prevent parents from maximizing available resources and interventions:

A lot of the clients we have are from disadvantaged backgrounds where they are probably been treated like they are not the experts in anything...and if they’ve been in abusive relationships, they probably have been told that they don’t know anything.

SP2

##### Dialogue Theme 2: Service Providers Miss Early Intervention Parenting Work

At the time of the dialogues, Family Services was transitioning to a service structure change, which resulted in changes to common family presentations (ie, families with more complex needs), meaning less time was available for early intervention work. Participants expressed that they valued hearing and seeing positive change and feedback from families and supporting them to feel empowered and missed these interactions with parents:

[W]e’re having a lot more child protection kind of clients ourselves, which might mean more crisis-type interventions, which is not really what we’re meant to be doing.

SP2

When you get that positive feedback and you feel like you’ve done some good, that’s the best.

SP2

##### Dialogue Theme 3: Limited Time and Human Resources Mean Limited Capacity for Learning New Technology

Participants acknowledged the benefits of technology in improving the efficiency of administrative requirements and improving ongoing engagement with families. They emphasized the importance of ensuring technological innovations were accessible for both families and service providers, due to both groups’ limited capacity for taking on challenging tasks, especially during COVID-19 lockdowns. They also discussed how technology should not replace aspects of service that require comprehensive observation:

[I]t needs to be user-friendly for our clients. Because particularly if they are very vulnerable...too much information, not [being] clear...it can be really hard.

SP1

If we can make things more flexible for families, there’s more chance that they’ll engage.

SP2

##### Key Design Considerations

Key insights gained from the dialogues included the need for fostering parent confidence and capacity to engage in parenting work and using simple and familiar technology to reduce additional parental and service provider stress. Thus, key design considerations included opportunities to build parents’ motivation and accessible technology.

#### Co-Design Workshop Series 1: Discovering the Design Space and Design Problems

##### Overview

Workshops (n*=*3) were conducted with 3 service providers from Family Services, 2 (67%) of whom had participated in the dialogues, and 1 (33%) had participated in an adjacent co-design project occurring within the community health service. Workshops were conducted between October and November 2021 using web-conferencing platforms (Zoom) due to COVID-19 restrictions. Each workshop lasted 1.5 hours, including two 5-minute breaks. The first author first introduced the workshop’s topic, then presented insights and reflections synthesized from each prior research activity. Participants were invited to respond to the researcher’s synthesis and share perceived *opportunities, considerations,* and *risks* regarding the workshop’s topic. Simple visuals to anchor discussions were provided while participants freely associated in the discussion. For the workshop focusing on technology-assisted intervention components, participants were provided with access to an existing technology-assisted intervention and were encouraged to think aloud while navigating the intervention.

A researcher (the first or the second author) recorded notes in situ during each workshop using Google Slides (Google LLC), including key quotes made by participants. All workshops were video and audio recorded via Zoom, and video recordings were reviewed by the first author to closely observe participants’ verbal and nonverbal responses and elaborate on notes and quotes taken in situ. These data were analyzed using the approach to deductive content analysis outlined by Elo and Kyngäs [44] and coded to correspond to categories of opportunities, considerations, and risks. Codes were then synthesized into key themes for sharing at the following workshop.

##### Design Problem 1: Simple Technology With Human Support

An opportunity for simple, hybrid models of intervention delivery was proposed by the participants. Participants discussed how their transition to using more technology-based methods of engaging with clients and completing work was relatively new and sudden in the context of working remotely due to COVID-19. They emphasized a need for technology-assisted components to be simple to navigate with “no guess work...it cannot be another thing I’m working on,” said SP3:

I was envisaging was a program that parents might complete in their own time, but can discuss the content with their support worker who checks in with them on a given basis. The support worker can provide ongoing encouragement and support in between contacts as well.

SP3

##### Design Problem 2: Parent Engagement Varies Across the Service Journey

Participants emphasized a key consideration around how parents’ engagement varies from client to client depending on factors, such as their readiness and the level of risk, and hence no discrete points on parents’ service journey would be more or less appropriate for a technology-assisted parenting intervention:

It’s more about understanding where a client’s head is at in their journey, rather than a specific point.

SP1

They also discussed factors observed as being associated with opportunities of parent readiness to engage in parenting work, such as expressing motivation or evidence of implementing changes in their parenting and insight into the role of parenting on children’s behavior*.* Building parents’ self-efficacy through reflecting the parents’ efforts was emphasized as important in helping parents recognize the progress they have made. Participants discussed the value their community health service places on empowering parents throughout their service journey to foster self-initiated change, as this can reduce overall service dependency, which in turn can reduce the waitlist burden:

We see many parents who want change but struggle with recognising or playing their role. Emphasising parents’ role in the change process early on is important to seeing that change.

SP2

##### Key Design Considerations

Key design considerations identified through these workshops reinforced the needs identified in the dialogues for accessible technology, as well as a need for ongoing availability and delivery methods that are highly adaptable so that the technology-assisted intervention can be deployed at any point on the Family Services service journey to align with parent readiness for engaging with such an intervention. A hybrid model was proposed, in which service providers’ role may be to support parental empowerment. Subsequent co-design workshops therefore focused on defining processes of empowerment that PaRK could feasibly support.

### Phase 2: Create a Minimum Viable Product

#### Co-Design Workshops Series 2: Defining Empowerment

##### Overview

Participants in series 2 workshops were the same participants from the dialogues (n=2), as the additional service provider from series 1 opted out due to a change in their role at the community health service. All workshops lasted 1.5 hours, with two 5-minute breaks. The first workshop started with the first author presenting a synthesis of data from prior workshops to provide rationale for the focus on empowerment. A definition of empowerment and related processes by Adams [45] was also introduced to frame activities: “The capacity of individuals to take control of their circumstances, exercise power, and achieve their own goals, and the processes by which they are able to help themselves [and others] to maximize the quality of their lives.” Processes were actionable approaches to overcoming barriers to individual empowerment and included *raising awareness, gaining skills, building confidence, self advocacy, networking,* and *taking action* [45]. Participants were invited to respond to the researcher’s data synthesis, centering the parenting intervention co-design to the concept of empowerment, and the proposed definition of empowerment. Participants were then invited to consider how these processes are currently practiced and how they would ideally be practiced, as well as ideas on how the ideal practice could be achieved based on their professional values.

All workshops were video and audio recorded via Zoom. During the workshop, the first author made live notes using an interactive board (Google Jamboard [Google LLC]) while service providers freely associated in the discussion on current and ideal practices related to processes of empowerment. Video recordings were reviewed by the first author to more closely observe participants’ verbal and nonverbal responses and elaborate on notes taken on the interactive board, as well as to identify key quotes. These data were examined, and additional notes were made on the interactive board by the first author to articulate apparent gaps and ideas to address these based on professional values. The broader research team (the first, second, and third authors) then discussed and synthesized findings on the boards to reach consensus on perceived needs and potential design implications for enhancing processes of empowerment.

##### Design Problem 3: Enhancing Empowerment

Participants agreed that empowerment was both a shared goal and a professional value that they would ideally like to strengthen in service provision:

Empowerment is definitely a shared goal for us and parents...that message can get lost a bit...The message should be clear: you’re going to be the one changing this, and we’re going to be supporting you.

SP2

**Figure 1:**
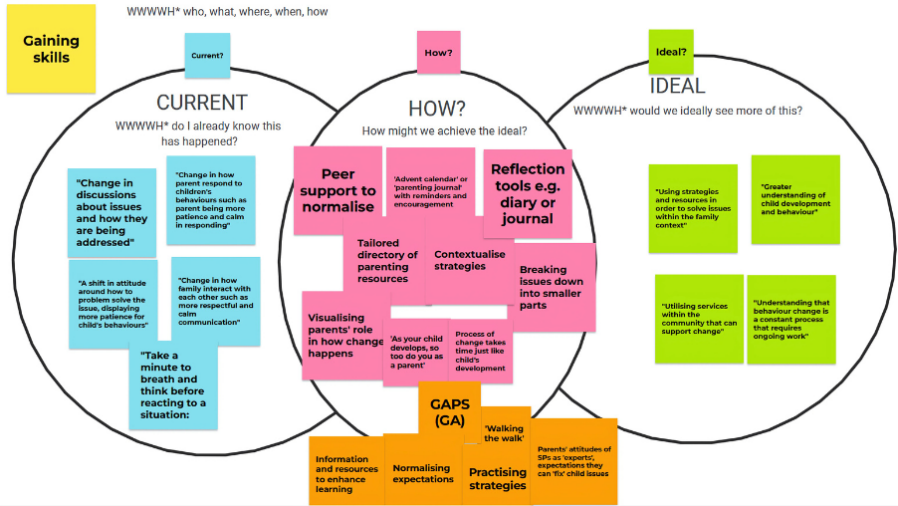
Snapshot of interactive whiteboard used in co-design workshops depicting service provider participants’ discussion points around current and ideal service practices, and first author’s proposed gaps between current and ideal service practices. (GA = Grace Aldridge, SP = Service Provider).

They discussed how building reflection and insight, patience and persistence, education, and peer support represented ideal practices to support parent empowerment. An example of the interactive boards depicting participants discussion points around current and ideal practices and the first author’s proposed gaps is found in Figure 1.

Key needs identified by the research team for enhancing processes of empowerment included the following: (1) Raising awareness—an opportunity during service contacts for parents and service providers to establish a clear and shared understanding of each other’s role in reaching care plan goals, (2) Gaining skills—resources and activities beyond a service contact to learn about and practice meaningful skills and strategies and normalize setbacks in service contacts, and (3) Building confidence—opportunities to notice and celebrate small-term and long-term changes.

Increasing opportunities for reflection emerged as both an underlying theme and priority gap that the PaRK co-design could feasibly address. The following quote from Adams [45] regarding the role of reflection as a process of empowerment was deeply considered: “By its nature, empowerment is a critical activity. Self-empowerment and self-advocacy necessitate reflexivity by the individual. Reflexivity involves using the impact of a situation or experience on oneself to help understanding and feed into future activity.”

##### Key Design Considerations

Embedding more opportunities and activities to encourage reflection to support the process of parental empowerment was identified as a final key design consideration. Service providers’ role in leveraging opportunities for reflection was deeply considered in producing intervention components that enable empowerment. Their role was considered because they are a consistent engagement resource for parents to pause and reflect with, motivated to empower parents, and trained in reflecting with others using collaborative approaches and building therapeutic alliances.

##### Final Design Implications and Intervention Components

The key design considerations were first consolidated into three design implications: (1) deliver intervention content with technology that is accessible, adaptable, and available on an ongoing basis; (2) adopt a hybrid approach using service providers to facilitate learning and reflection and enhance parental empowerment over intervention content; and (3) leverage existing skills and service elements to embed intervention delivery into service providers’ existing practice.

The intervention components *podcasts* and *microcoaching* were developed to meet design implication needs.

Podcasts were developed to deliver PaRK’s evidence-based content. Podcasts are accessible as they require minimal media literacy; they are adaptable as content can be broken down into smaller “bite-sized” chunks or modules and sent depending on parents’ readiness and preference and they facilitate continuous learning and reflection with playback features and ongoing availability. In total, 3 out of a possible 12 topics from the original PaRK intervention were nominated by recruited service providers to be redesigned into trial podcasts, based on the parenting issues or concerns most commonly encountered with parents in their service. They were *helping your child manage emotions, establishing rules and consequences,* and *managing conflict in the home.* The design and development of these podcasts comprised a separate research project. It was informed by media effects theory, coupled with an “object-based media” approach [46,47].

Microcoaching was proposed to complement the process of acquiring knowledge from the podcasts, leverage the “human element” of service providers to facilitate reflection, and meet the need for embedding more opportunities for reflection into existing service provision practices. Coaching can be defined as a learning process tailored to the learner’s needs through strengthening existing capacities for growth, characterized by collaboration, reflection, and centering on goals [48]. Microcoaching refers to using very short “capsules” of coaching built into existing service contacts between parents and service providers, designed to meet goals that are specific and feasible to achieve in short timeframes. In this way, microcoaching promotes parent reflexivity, gaining skills and confidence, all of which support the process of empowerment [45]. The design and development of the microcoaching component are reported in the Co-Design Workshop Series 3: Developing a Prototype for Microcoaching section.

**Figure 2:**
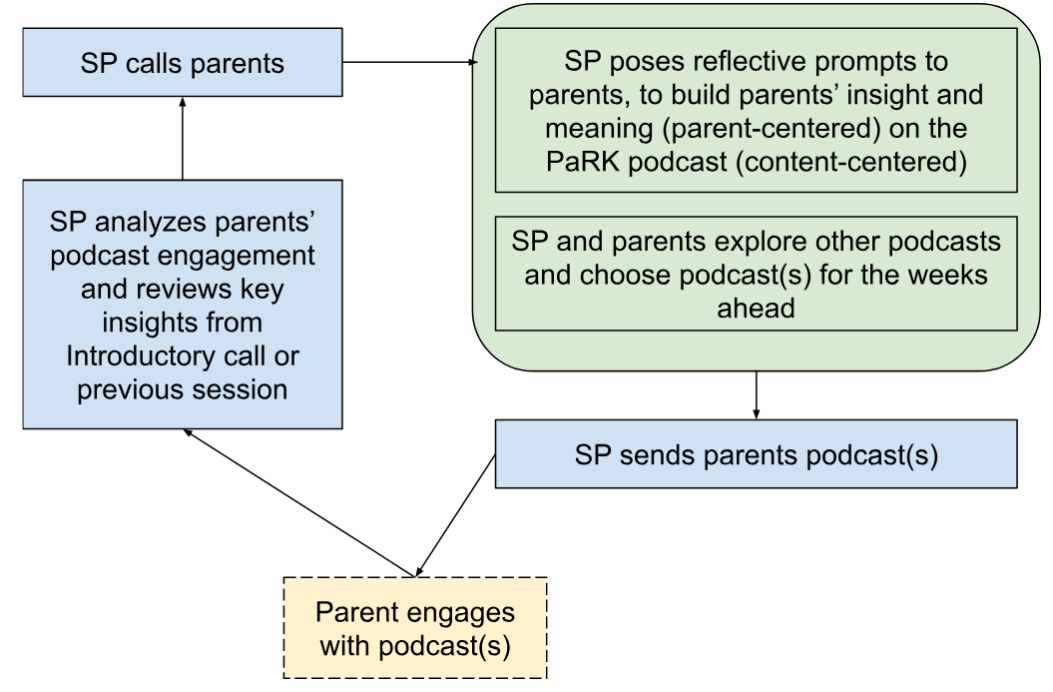
Proposed flow of the co-designed parenting intervention delivery (podcasts and microcoaching). PaRK: Parenting Resilient Kids; SP: service provider.

Figure 2 depicts the proposed intervention delivery flow for the co-designed podcasts and microcoaching.

#### Co-Design Workshops Series 3: Developing a Prototype for Microcoaching

##### Overview

Participants were the same service providers who participated in the co-design workshops and the dialogues (n=2). The first author developed prototype components between workshops, based on the insights and feedback generated during the prior workshop. The first workshop focused on exploring how service providers currently practice reflection with parents so that these practices could be leveraged in microcoaching components. Reflection was defined as “looking for relationships between pieces of experience or knowledge, evidence of cycles of interpreting and questioning, consideration of different explanations/hypotheses and other points of view*.*” [49]. The focus of each subsequent workshop (n=2) was 2-fold: role-playing microcoaching prototypes and providing direct, experience-based feedback. Feedback discussions were semistructured, guided by the “4 Ls” (what was liked, what was lacking, what was learned, and what was longed for).

The first author recorded notes in situ during each workshop. All workshops were video and audio recorded, and video recordings were reviewed by the first author to observe service providers’ verbal and nonverbal responses more closely and elaborate on notes taken in situ. Deductive content analysis was used to code data according to the “4 Ls.” Data were examined, and additional notes to articulate apparent gaps and ideas to address these were added by the first author and accounted for in the subsequent microcoaching prototype.

##### The Microcoaching Prototype

**Figure 3:**
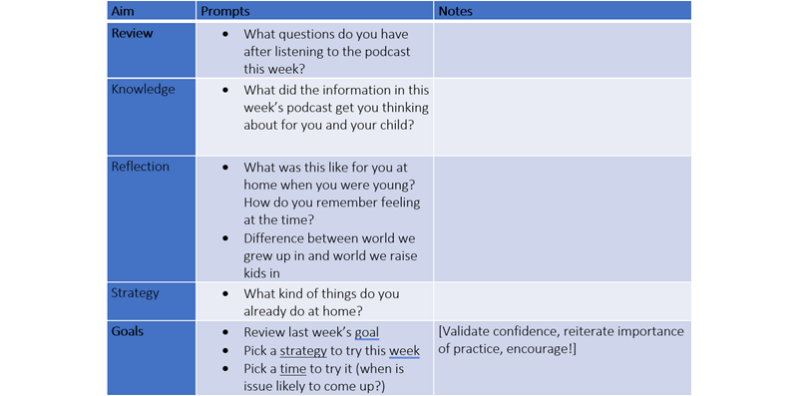
Sample of Parenting Resilient Kids–Lite’s microcoaching session plan and note-taking template for service providers.

**Figure 4:**
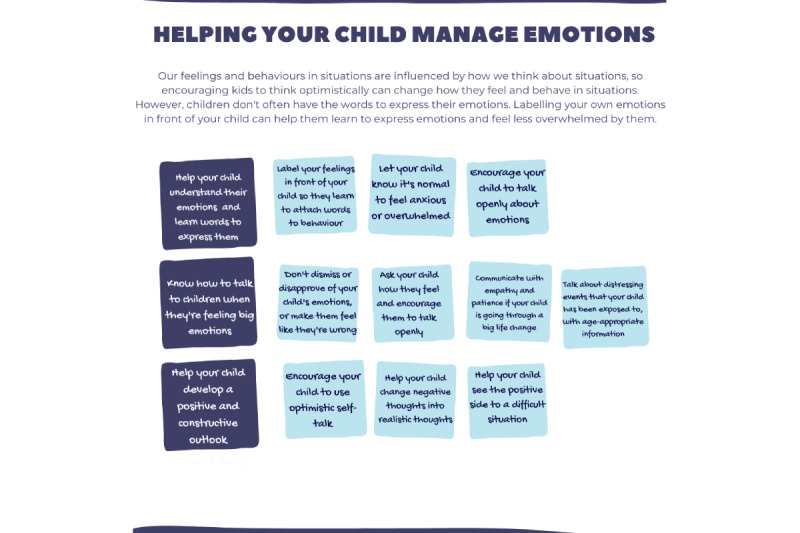
Parenting Resilient Kids–Lite “cheat sheets” for parents and service providers summarizing the strategies presented in each podcast.

**Figure 5:**
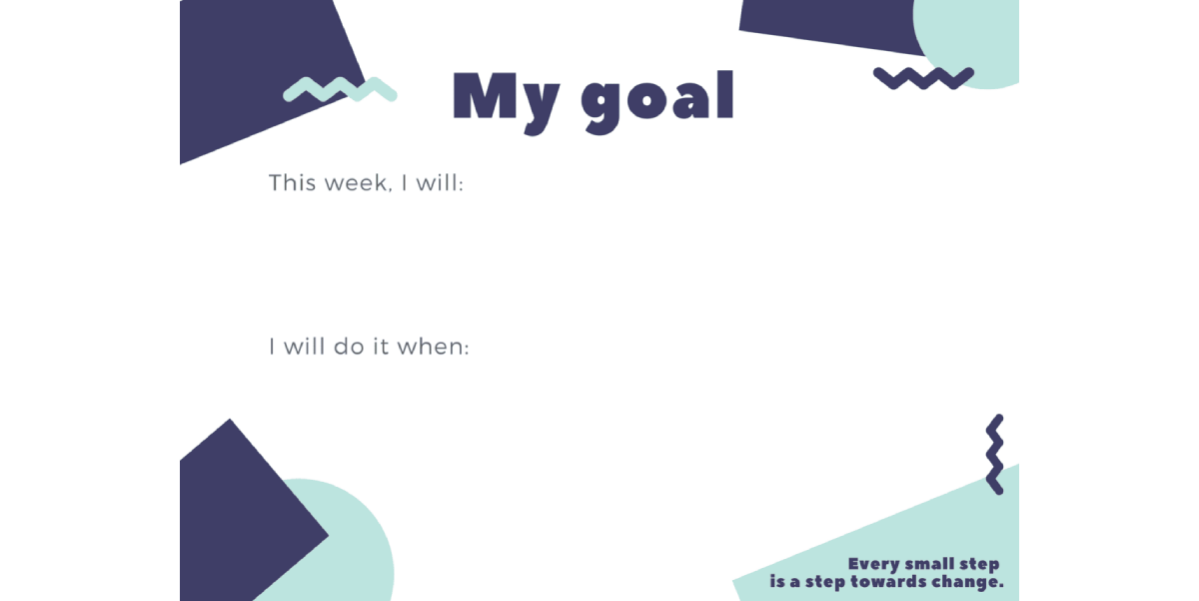
Parenting Resilient Kids–Lite “goal-setting” card for parents to list their goals and keep with them between sessions.

The microcoaching “minimum viable product” consisted of semistructured microcoaching session plans with note-taking templates attached (Figure 3) and tangible artifacts to build shared understandings and support goal attainment, including a colorful and easy-to-read “cheat sheet” presenting each podcast’s evidence-based strategies and a goal-setting card for parents to refer to between microcoaching sessions (Figures 4 and 5). Full details of the PaRK-Lite’s microcoaching components, including service touchpoints, functions and goals, and descriptions of each activity, can be found in Multimedia Appendix 2.

A manual for service providers outlining the entire process of engaging parents with PaRK-Lite was also developed along with a training session to actively practice the process in a step-by-step manner. All material was uploaded into a dedicated Microsoft Teams (Microsoft Corp) channel, a platform currently used by all staff at the community health service. The redesigned PaRK was subsequently named PaRK-Lite, capturing the adaptations’ aim to deliver the PaRK intervention in a lighter-touch format.

### Phase 3: Validate the Prototype’s Design

#### Overview

A total of 7 parents expressed interest in participating, of which 6 (86%) provided consent and completed the set-up session. In total, 2 (29%) parents requested to withdraw due to family emergencies, and 1 (14%) parent could not be reached for follow-up. In total, 3 (43%) parents completed at least 1 microcoaching session, so their data were included for analysis. In addition, 2 (67%) of these 3 parents completed the feedback interview, as 1 (33%) could not be reached for follow-up. Participant demographic information is presented in Table 1.

The first author, who is a provisional psychologist with clinical experience working in family violence and public health settings, delivered the PaRK-Lite prototype (podcasts and microcoaching) to recruited parents between June and August 2023 and conducted feedback interviews between July and August 2023. All microcoaching sessions and feedback interviews were completed via telephone and were recorded using a dictaphone. Interviews followed a semistructured format using an interview guide developed by the first and second authors designed to inquire about parents’ experience of engaging with PaRK-Lite and evaluate its key design considerations (Multimedia Appendix 3). It was reviewed by a peer researcher for content validity and to ensure the language was sufficiently simple and appropriate. Field notes were written during and after each interview to support reflection and interpretation of the interview transcript. Credibility was achieved through peer checking and triangulation of field notes. Interviews were transcribed by a third-party transcription service and checked by the first author for accuracy. Data collection and analysis occurred concurrently.

The first author collected parents’ podcast engagement data from a web-based podcasting platform (Transistor [Transistor.fm. Broadcast Media Production and Distribution) between sessions using a purpose-built web app that provided deidentified information on episodes opened, completed, and playback functions used. Microcoaching session recordings and feedback interview data were analyzed using thematic analysis. The 3 design implications (refer to the Final Design Implications and Intervention Components section) informed start list categories under which parent feedback data were coded inductively by the first author, following the six-step process as described by Braun and Clarke [50]: (1) familiarization with the data, (2) coding, (3) generating initial themes, (4) renewing themes, (5) defining and renaming themes, and (6) writing up. NVivo (Lumivero) data analysis software was used to code data. Codes were discussed between the first and second authors to ensure clarity of meaning. Following the coding process, initial themes for each design implication were constructed and discussed with the broader research team until agreement was reached.

**Table 1:** Participant demographics of parents receiving Parenting Resilient Kids–Lite prototype (N=3).

			Value
Age (y), mean (SD; range)			34.6 (3.7; 30-39)
**Gender, n (%)**			
	Men	1 (33)	
	Women	2 (67)	
**Born outside Australia, n (%)**			
	Yes	1 (33)	
	No	2 (67)	
**Language other than English spoken at home, n (%)**			
	Yes	1 (33)	
	No	2 (67)	
**Number of children, n (%)**			
	3	2 (67)	
	4	1 (33)	
Age of children (y), mean (SD; range)			7 (1.6; 5-9)
**Child or children with mental-health diagnosis, n (%)**			
	Yes	2 (67)	
	No	1 (33)	
**Service used at the community health service, n (%)**			
	Counseling and well-being	1 (33)	
	Family Services	1 (33)	
	Health services	1 (33)	
	Medical services	1 (33)	
	Other (not reported)	1 (33)	
**Concession or health care card holder** ^a^ **, n (%)**			
	Yes	1 (33)	
	No	2 (67)	
**Educational level, n (%)**			
	Secondary	1 (33)	
	Tertiary	2 (67)	
**Family structure, n (%)**			
	Nuclear family	2 (67)	
	Single parent	1 (33)	
**Experience of life challenges, n (%)**			
	Yes	1 (33)	
	No	2 (67)	

^a^Provides recipients of government benefit schemes and low-income earners with access to cheaper prescription medicines and health care services.

#### Engagement Data

*Setup* call durations ranged from 9 to 18 (mean 14.6, SD 2.75) minutes. *Microcoaching* call durations ranged from 7 to 40 (mean 22.37, SD 10.96) minutes. Longer calls were due to 1 parent spontaneously providing feedback on the podcasts in addition to the microcoaching. All parents (3/3, 100%) expressed an interest in receiving all episodes from all 3 podcast topics (with all parents opting to start with *helping your child manage* emotions); however, 2 (67%) parents chose to receive 1 podcast topic (12 episodes) and 1 (33%) parent chose to receive all 3 topics (36 episodes). Adherence to selected podcasts ranged from 42% (5/12) to 81% (29/36; mean 65%, SD 17.15%). Podcasts replayed by parents ranged from 6% (2/36) to 58% (7/12; mean 23%, SD 24.75%). Feedback interviews averaged 52.67 (SD 0.36) minutes.

#### Qualitative Data

Microcoaching sessions and feedback interview transcripts revealed themes pertaining to how PaRK-Lite’s design met its intended purpose and parents’ needs, as well as themes indicating emergent needs that the next iteration of PaRK-Lite could address to support parents’ engagement. A novel theme relating to engaging children with the podcasts also emerged.

#### Technology (Podcast) Feedback

##### Accessibility

Participants described how flexible delivery features (such as, tailored delivery of reminders and the podcast’s playback features), relatable podcast scenarios, and relevant topics helped the content and delivery of PaRK-Lite feel accessible:

The way you’ve structured your modules and the way you guys are delivering them, they’re very engaging as opposed to what they [another existing web-based parenting program] have got.

P21

##### Adaptability

The collaborative focus of microcoaching sessions and flexibility around session scheduling, having podcast chunks sent at specific times in the day depending on changing schedules, and the ability to progress at their preferred pace helped participants feel that PaRK-Lite was highly adaptable and tailored to their needs, readiness, and personal circumstances:

The flexibility and understanding around it, I guess. That was really, really good.

P18

##### Continuity (Ongoing)

Parents expressed an intention to listen to the podcasts in the future to either refresh their memory of strategies previously discussed or practiced or to facilitate self-guided learning as their family’s situations and needs change or emerge:

It’s definitely something that I’d probably go back to in case—when I just need a little reminder of if I’m doing okay.

P18

Emerging needs expressed by participants that future iterations can address included tips to centralize chunks and reminders sent by the coach so that they are all in one place to help parents track their progress, reducing the amount of slang and broadening the podcast’s genre to appeal to parents from culturally diverse backgrounds to enhance accessibility, further resources for navigating conversations with age-appropriate information, and other parenting support designed for parents of children with diagnosed mental health or behavioral problems to support ongoing learning.

#### Empowerment Through Reflection (Microcoaching) Feedback

Participants reported that the use of everyday scenarios and dialogues between parents and children to illustrate parenting strategies was highly effective for prompting reflection and insight into the different perspectives between themselves and their children:

I never realized how it sort of feels for the kids. So that that podcast did feel like that day: “Oh, they might must have been going through some big emotions when we were doing that.” So that was sort of a provoking one.

P21

The use of probing questions and paraphrasing by the coach during microcoaching was reported as helpful in both contextualizing podcast content and appreciating the value of their own observations and experience. Common insights included greater awareness of what may be triggering their child’s behavior, a broadened understanding of their own or their child’s perspective, strategies that are most relevant to the changes they are trying to enact, and opportunities in which trying out these strategies might be most effective:

They have been great. I think they just set the right perspective to things as well...helped me understand...and sort of made me think.

P21

Participants reported goal setting supported them to enact strategies and reflect on the outcome with the coach. Both participants opted to enact the strategy of being curious rather than dismissive and asking their child how they feel if they are expressing big emotions. One parent reported their child had since become more “relaxed” in talking about their emotions, while the other reported their child was initially surprised and took some time to open up. Both participants reported feeling accomplished as a result, which increased their sense of self-efficacy about implementing strategies in the future, although mild hesitance was expressed about how situational factors might influence how effectively they can implement strategies:

I’m feeling pretty confident about that change now, especially after last night’s ordeal. When I confronted that in a calmer way than what I expected I would.

P18

Emerging needs expressed by participants that future iterations can address included the following: resources for writing down reflections and insights to support knowledge acquisition, further resources for contextualizing podcast content to their family’s needs, and practicing strategies through modeling (ie, through watching videos) or role playing during microcoaching.

#### Embedding Into Existing Services

Participants reported several factors that would enhance their engagement with PaRK-Lite if embedded within the community health service. First, participants reported that PaRK-Lite should be presented as an option rather than a recommendation, as being recommended to engage with interventions could result in feeling pressured to engage, and the subsequent stress would likely reduce their engagement and further negatively impact their parenting:

If this were something that I was expected to do...it could impact my parenting in quite a negative way...because it would stress me out more.

P18

Second, they reported that service providers’ expectations of parents’ engagement with PaRK-Lite should be flexible and collaboratively structured, as this was a highly valued aspect of their experience and contributed to them feeling less pressure and more empowered:

It’s flexible, it’s within your own time, there’s no real expectation...I think that would make me feel more inclined to keep going.

P18

#### Emergent Theme: Involving Children

Both parents discussed how they either did, or intended to, listen to the podcasts with their children to facilitate their children’s learning about the podcasts’ topics and to serve as an exercise to build mutual understanding and connection:

Having kids sit through these podcasts helps also that they understand what the concept and the reasoning behind them is.

P21

## Discussion

### Principal Findings

This co-design study aimed to innovate a community health service with technology-assisted parenting support for families of children who experience adversity. Our key findings are design implications regarding the needs and preferences expressed by service providers and parents. First, they expressed that technology-based innovations that are accessible, adaptable, and offer ongoing availability promote engagement with delivering or receiving the intervention, respectively. Second, strengthening processes of parental empowerment enhances engagement with parenting support and promotes changes in parenting. Finally, embedding technology-based innovations as seamlessly as possible into existing service provision practices reduces additional workload burden for service providers and appeals to parents as long as engagement is optional. These findings will be detailed and discussed in the following sections with reference to existing literature.

### Designing for Technology-Assisted Service Provision

The first design implication focuses on the technological needs and literacy in this study’s Family Services context. Accessibility, adaptability, and continuity were deemed critical for integrating technology into the Family Services context, because the need to learn or set up technology was likely to lead to disengagement given the high-needs and low-resource context. Service providers also expressed that the human element and connection should not be replaced. These findings echo the broader literature in that acceptance of technology will likely be enhanced if technology does not significantly interfere with building engagement and rapport and is easy to use or requires low technological literacy [25,51]. We therefore suggest that including practitioner attitudes and skills in using technology is a key factor to consider for designing and implementing technology-assisted support in health services.

Previous research has identified that interventions with complex language can lead parents with differing literacy levels or linguistic diversity to doubt their ability to complete an intervention [21] and that audio or video formats are preferred by intervention users [52]. The use of sports commentary as a genre, humor, and everyday scenarios to illustrate parenting strategies in the podcasts enhanced parents’ self-reported relatability to the information presented and subsequent engagement with the content. However, reducing instances of slang and broadening the genre and narrative style to appeal to a broader range of parents from different cultural backgrounds is suggested for both future adaptations of the podcast and future research.

Prior research has also indicated parents experiencing socioeconomic disadvantage often face multiple responsibilities to manage socioeconomic disadvantage [53] and prefer briefer intervention durations so they can accommodate intervention access and engagement between these responsibilities [21]. This was echoed as a need by service providers in phase 1 of this study. In response, we developed podcasts that delivered short and focused content in small, “bite-sized” chunks. Breaking down complex topics into smaller units can help parents absorb information and can facilitate parents’ engagement with the content in small increments if needed. This structure can also reduce feeling overwhelmed by information, which has been reported as a barrier to engagement by service providers in this study and by parents in prior research [54]. Parents in this study reported that the ability to consume chunks of evidence-based information at preferred times was effective in supporting them to engage, reflected in the overall high completion rates.

The “chunk” structure of the podcasts also allows parents to choose the amount of content and pace of engagement, which responds to service providers’ need for adaptable technology that they can tailor to meet the specific needs of parents at any point in their service journey. Building in adaptability can influence acceptance of technology, as it facilitates the innovation deliverer’s perceived competence with delivery [25] and indeed facilitates their implementation of the innovation [21]. The microcoaching also supported tailoring by allowing parents to establish the session focus depending on their needs and circumstances, which was in turn reported by parents in this study as a highly valued aspect of their experience.

Ongoing access to support is vital for maintaining engagement and fostering long-term change [55], especially in contexts where behavioral change can be a long-term process due to the many stressors and challenges faced by the parents accessing Family Services. Designing for continuity involves ensuring that the technology-based services are available and accessible over an extended period [56]. Parents in this study reported that features of PaRK-Lite designed for continuity (podcasts and written artifacts) facilitated self-guided learning and contributed to their sense of self-efficacy about navigating their parenting in the future.

### Empowering Parents Through a Tailored, Hybrid Approach

The second design implication focuses on empowering parents to engage with services and interventions. Prior research has indeed demonstrated that parents’ everyday stressors and concerns about parenting are negatively associated with parental empowerment [57], which in turn has been positively associated with parental engagement [58]. To address this, we explored how PaRK’s redesign could facilitate opportunities for parents to learn and reflect on their parenting, as self-empowerment necessitates reflexivity [45].

Parental empowerment has been conceptualized as “a process through which families can access knowledge, skills, and resources that enable them to gain positive control over their lives” [59]. Service providers expressed that integrating evidence-based tools to outsource some parenting work would enhance parental empowerment in the long term for reducing service dependency and waitlist burden. We responded to this by developing entertaining podcasts and accompanying concrete artifacts. Parents in this study reported that the humor and relatability of the everyday scenarios and dialogues between parents and children in the podcasts were critical in their process of learning and prompting reflexivity, thus enhancing their process of self-empowerment. In fact, parents expressed an emerging need for access to additional and further resources to continue their learning, which future iterations of PaRK-Lite can respond to.

Because service providers expressed that the human element and connection should not be replaced, a hybrid approach to intervention delivery was developed, with the “human element” facilitating opportunities for reflecting on parenting between parents and service providers. Hybrid approaches to intervention delivery have emerged as a promising model in various educational and professional settings [60]. For instance, adaptive algorithms and analytics can enable tailored delivery of content, while the presence of coaches or service providers fosters a supportive and collaborative environment that promotes active discussion, questioning, and feedback to enhance critical thinking, engagement, and a sense of belonging [61]. Human support can enhance adherence to digital interventions [8,62,63], possibly because exploring and addressing parents’ concerns is important in building parents’ motivation to engage with parenting interventions [64]. In this study, parents reported that their contact with a coach, who supported them to appreciate the value of their insight and feel empowered by their knowledge and experience, contributed to changes in their parenting and increased self-efficacy around implementing parenting strategies in the future. Overall, the hybrid approach to intervention delivery offers a rich and comprehensive learning experience that maximizes the benefits of both technology-based and human elements.

### Embedding Innovations Into Existing Practices

The third design implication focuses on embedding the co-designed innovation into existing practices. Prior research has suggested that innovation adoption is more likely when the innovation is perceived as meaningful and valuable and involves fewer changes to existing practices [24,65]. We thus leveraged existing service practices and adapted existing service elements (ie, the “soft peripheries”) to facilitate adoption, implementation, and scalability of the evidence-based intervention (ie, the “hard cores”).

The microcoaching component, which was the “human” element of PaRK-Lite’s hybrid design, intended to leverage service providers’ existing coaching skills (such as active listening, communication skills, and building motivation [48]) and enhance parental empowerment. A “coach” approach strengthens parental empowerment, as practitioners take a facilitative approach rather than an authoritative approach to engaging with parents, share power, and encourage independence [45]. This approach is consistent with the Supportive Accountability model, which predicts intervention engagement can be enhanced when eHealth interventions include a coach who is perceived as trustworthy and benevolent, willing to involve people in defining goals and expectations, and frames performance monitoring as devoid of negative consequences [66]. Prior research has demonstrated that service users appreciate practitioners’ willingness to engage in conversations that foster empathy [67] and that embedding relational strategies, such as microcoaching, into existing points of contact is especially important for enhancing underserved communities’ trust and engagement with services [68]. Overall, microcoaching exemplifies how existing staff resources can be leveraged to promote the use of evidence-based parenting support by both staff delivering such support, and parents receiving such support.

PaRK-Lite’s hybrid design included light-touch requirements to ensure that it could be embedded into existing service elements of Family Services. The brief intervention duration of PaRK-Lite complemented the short-term interventions offered during the “active holding response” service element and can further help reduce frustration associated with being on waitlists [69]. The self-directed learning and opportunities for reflection through microcoaching were also consistent with the underlying goal of “medium-term casework” to empower parents. Parents in this study expressed that if embedded, PaRK-Lite should be presented as a universal option rather than a targeted recommendation.

### Limitations

First, our co-design methodology involved understanding contextual needs and the design space for adaptations through service providers only, as COVID-19 significantly impacted our ability to reach parents who were experiencing additional stress due to lockdowns. Similar recruitment difficulties have been documented elsewhere [70]. While our method is consistent with recommendations from prior research [21] and may represent an efficient method for reducing the time asked of participants while upholding principles of co-design, it did not permit an authentic exploration and integration of parents’ needs from the outset. Such views may have shaped the initial design of PaRK-Lite’s prototype, particularly with regard to the podcast’s genre and narration style. Consistent with the Double Diamond model [37], PaRK-Lite is being designed iteratively so that as limitations emerge, subsequent iterations can address them until the intervention is deemed ready for larger-scale implementation and evaluation. Hence, the next iteration of PaRK-Lite can prioritize exploring and testing other podcast genres and narration styles. We encourage future research interested in co-designing hybrid parenting interventions to adopt an iterative approach to ensure limitations are addressed before larger-scale or real-world translation. Second, the participant sample size in both phases 1 and 2 was small. A small sample size is considered appropriate for a small-scale, in-depth, and case-oriented approach to analysis [39,40], and we intended for our findings to provide the literature with an example of the utility of this methodological approach. We also ensured that recruited service providers were representative of touchpoints with parents on the Family Services journey, and recruited parents came from very different cultural backgrounds regarding gender, religion, and family structure. However, integrating a wider range of service provider and parent views may have shaped PaRK-Lite’s initial design, especially from parents with lower motivation to remain engaged and provide feedback compared with the parents who participated in this study. Furthermore, although our findings are overall thematically consistent with prior research, this study’s small sample size limits the generalizability of its findings to other contexts and the robustness of its thematic contribution to the literature. Third, PaRK-Lite was delivered to parents by the first author, who also contributed to PaRK-Lite’s co-design and conducted parent feedback interviews, rather than a service provider. This may have influenced how parents’ experience and feedback of PaRK-Lite was interpreted and presented and introduced potential gaps or biases in understanding the feasibility for service providers to deliver PaRK-Lite in its current format. Having service providers deliver PaRK-Lite at a small scale is thus a priority for the next phase of its iterative development process. The Double Diamond framework for design emphasizes iterative development and is not intended to be adhered to in a discrete or linear manner [37]. This study represents the first design iteration, and we intend to integrate these limitations along with parents’ feedback on PaRK-Lite’s initial design into its next iteration. We also intend to evaluate potential organizational barriers and facilitators to implementing PaRK-Lite and develop strategies to support its implementation based on that. We believe this level of formative research is necessary to reduce foreseeable logistic complexities and facilitate translation into real-world settings, as this in turn may facilitate conducting a larger-scale, real-world evaluation of PaRK-Lite.

### Directions for Future Research

Findings from this study provide strong support for a hybrid approach to delivering technology-assisted parenting intervention components. To facilitate translation into the Family Services context, we identified existing service elements wherein families’ unique circumstances (such as family dynamics and socioeconomic resources or constraints) are considered as part of a collaborative and tailored intervention planning process (ie, the Family Services Care Plan). We suggest that, in future, researchers interested in advancing hybrid parenting interventions use the Double Diamond approach to continue exploring and refining how microcoaching can be translated into a given real-world setting and complement technology-assisted methods of delivering evidence-based parenting support. Specifically, we suggest involving service providers in this process as they possess specialized knowledge into existing service elements and existing family-specific practices that may facilitate translating microcoaching into real-world settings and meet the diverse needs that families present with. We also suggest evaluating relevant implementation outcomes and intervention outcomes to understand how such elements and practices enhance engagement between parents of children experiencing adversity and services that support these parents.

A novel finding that emerged from this study was related to parents spontaneously involving their children in their engagement with the podcasts, suggesting PaRK-Lite’s design appeared to empower parents to creatively engage with the content to facilitate reflection and understanding between themselves and their children. This finding is also consistent with prior research, which has found preliminary evidence for improved parent-child interactions following engagement with digital parenting interventions [71,72] and also that fathers prefer web-based parenting interventions that involve their adolescent child with them [73]. In future, researchers interested in enhancing interested in enhancing parents’ engagement with technology-assisted parenting interventions may thus consider involving both parents and children as co-designers to explore how such interventions can be used to facilitate both their learning and sense of connection and understanding of each other and in turn, parents’ engagement with and benefits from these interventions.

### Conclusions

In conclusion, findings from this study support the key role that service providers play in early phases of innovation, as they possess both specialized knowledge about the contextual structures that may be innovated as well as sufficient insight into the lived experience of parent clients to design an appropriate prototype that overall meet parents’ needs. Our findings suggest that empowering parents by embedding reflective practice and accessible and adaptable technology was key to designing an appropriate technology-assisted parenting intervention for parents of children experiencing adversity. Researchers, practitioners, and designers in the field of human-computer interaction and health service design can consider our methods and findings in creating engaging interventions that have a positive impact on the well-being of children and families.

## Appendix

Multimedia Appendix 1 Co-design methods and aims.

Multimedia Appendix 2 Details of microcoaching components.

Multimedia Appendix 3 Parent interview schedule.

## Abbreviations

ACE: adverse childhood experience
PaRK: Parenting Resilient Kids

## Data Availability

The datasets generated during and analyzed during this study are not publicly available to protect participant privacy and anonymity, but are available in deidentified form from the corresponding author on reasonable request.
