# Supplementary material for: Embedding Technology-Assisted Parenting Interventions in Real-World Settings to Empower Parents of Children With Adverse Childhood Experiences: Co-Design Study
Source: JMIR Form Res. 2024 Nov 22;8:e55639. doi: 10.2196/55639 (PMC11624457; doi:10.2196/55639)
Supplement: Multimedia Appendix 1 [file formative_v8i1e55639_app1.docx]

## **Supplementary File 1**

**Co-design methods and aims**

| **Table 1.** Co-design methods and aims | |
| --- | --- |
| ***Method*** | ***Aims*** |
| **Phase 1:**  Identify design problems and design spaces for a technology-assisted parenting program through understanding service providers’ experience of contextual structures, systems and processes (i.e. ‘soft peripheries’). | |
| Dialogues  (*Discover*) | Gain a deep understanding of service providers’ experience of providing services to parents and using technology within Family Services, and the value and meaning service providers ascribe to their experience of working with parents.  Foster a relational approach to communication between the service provider and the first author. |
|  |  |
| Co-design workshop series 1: Discovering the design space and design problems  (*Discover*) | Understand the design space by identifying appropriate touchpoints in the service journey for a technology-assisted parenting program, and discussing potential design problems  Discuss how specific technology-assisted components should be tailored for Family Services and why. |
| **Phase 2:**  Create a minimum viable product that fits in the design space and can potentially address the design problem | |
| Co-design workshop series 2: Defining the design problem and space to address  (*Define*) | Identify gaps between current and ideal practices relating to identified design problems, by discussing how service providers would currently know the design problem has been resolved, and which processes supporting that could be strengthened. |
| Co-design workshop series 3: Prototype development (*Develop*) | Iteratively develop technology-assisted parenting program components by presenting and enacting proposed program components in co-design workshops then refining components based on insights and feedback. |
| **Phase 3:**  Validate the prototype’s design | |
| Prototype pilot  (*Deliver*) | Provide target end-users with the prototype program at a small-scale |
| Feedback interviews  (*Develop*) | Conduct qualitative interviews to understand if and how the prototype’s program met parents’ needs and preferences for a technology-assisted parenting program, and if further adaptations are needed. |
