# Supplementary material for: Embedding Technology-Assisted Parenting Interventions in Real-World Settings to Empower Parents of Children With Adverse Childhood Experiences: Co-Design Study
Source: JMIR Form Res. 2024 Nov 22;8:e55639. doi: 10.2196/55639 (PMC11624457; doi:10.2196/55639)
Supplement: Multimedia Appendix 2 [file formative_v8i1e55639_app2.docx]

## **Supplementary File 2**

**Details of micro-coaching components**

**Table 1.** Details of micro-coaching components

| **Component** | **Touchpoint** | **Function and/or goal** | **Description of activity** |
| --- | --- | --- | --- |
| **Introduction sessions** | During an existing service contact, to be assessed by service provider based on parent eligibility and readiness | Make parents aware of PaRK-Lite, invite them to receive PaRK-Lite, and prevent ‘information overload’ and disengagement by providing parents with an opportunity to consider their decision. | The service provider introduces PaRK-Lite to parents by describing the program’s topics, the podcast and micro-coaching components, and intended benefits. In the optional additional call, for parents who wish to consider their decision, the service provider uses motivational interviewing techniques to guide parents towards making their decision about accepting or declining the invitation. |
| **Set-up session** | During an existing service contact, following Introduction session(s). | Set up the PaRK-Lite program with parents, both in terms of logistics and practicalities as well as their motivation for engaging to frame future micro-coaching sessions. | The service provider opens the session by asking the parent what their hopes are for their child, and podcast topics are selected accordingly. The service provider also discusses when the podcasts will be sent to parents and when micro-coaching sessions will occur. Choices are summarised and hopes are affirmed. |
| **Micro-coaching session** | During an existing service contact, following Set-up session | Understand and trouble-shoot any barriers to engagement if podcasts are not listened to, or reflect on the podcast content towards trying out new parenting strategies. | If podcasts are not listened to, service providers explore validate competing demands, while gently reiterating parents’ role in making desired change. If podcasts are listened to, service providers invite parents to offer their main impressions to lead the session from the outset. The rest of the session is focused on encouraging parents’ personal reflection on the podcast’s content and their own parenting experiences. Depending on service provider preference, they may refer to general or specific micro-coaching prompts to support the discussion. Goals for trying new strategies are collaboratively reviewed and re-set. |
| **Session note templates** | Used during contacts between service providers and parents | Provide service providers with material to support structuring the sessions and record session content and impressions. | Service providers copy a session note template into a parent’s folder prior to each session. Service providers then enter notes either during or after the micro-coaching session directly into the template. Session notes are formatted so that space for notes is provided next to each prompt. |
| **Cheat sheet and goal cards** | Used during contacts between service providers and parents or in parents’ own time. | Provide parents and service providers with tangible artefacts to support building a shared understanding and recall of strategies and goals. | The generic ‘cheat sheets’ map the core evidence-based strategies from each module, either electronically or on paper. They can be used in micro-coaching sessions as a reference or grounding point for both parents and service providers, or between sessions by parents as a visual aid to complement podcast content. The ‘goal cards’ state the parents’ weekly goal for trying out a new strategy, and includes details of the agreed times to try the strategy, either electronically or on paper. They can be used in micro-coaching sessions as a “joint attention point” (SP1) for both parents and service providers, and between sessions by parents to support recall of strategy goal plans. |
| **Manual** | During training session and throughout PaRK-Lite delivery | Provides a brief overview of PaRK-Lite’s intended purpose, practical steps to complete micro-coaching (the ‘*what, when and how*’), and embedded session plan and note templates with hyperlinks to the documents’ and artefacts’ locations. | The Manual is a written document. It will be available to all service providers via Microsoft Teams. Service providers will be encouraged to familiarise themselves with the Manual prior to the PaRK-Lite training session. Service providers can then refer to the Manual throughout delivery of PaRK-Lite as needed. |
| **Training session** | Prior to service providers’ delivering PaRK-Lite, at the discretion of each team or service. | Provide service providers with an opportunity to: practise each step of the micro-coaching, reflect on their professional values and PaRK-Lite’s purpose, reflect on their perceived self-efficacy with delivering PaRK-Lite, and modify any processes or procedures to improve its fit with existing processes within a given service. | The training session aims to practically walk service providers through the manual. A facilitator will lead service providers through the training session using a PowerPoint presentation.  The training session will open with asking service providers to reflect on why they chose this profession, then review the key insights from the co-design process that informed the development of PaRK-Lite. A visual depicting the entire flow of program delivery is then provided, highlighting parents’ and service providers’ role in the flow.  Consistent with the Manual, the ‘*what, when and how*’ of each micro-coaching session (Introduction, Set-up, Micro-coaching) is reviewed. Service providers are then asked to build a parent persona, and role play each micro-coaching session with a colleague. Service providers are asked to locate micro-coaching material in Microsoft Teams before and during the role play, and practice entering session notes. Space for open discussion, reflection and feedback about the role play experience is then opened.  The training session is closed by asking service providers to rate their confidence in: understanding the PaRK-Lite process, knowing where to find the micro-coaching material, and familiarity with the micro-coaching session content. A discussion about how ratings could be raised is then opened, and the group may agree to modify micro-coaching processes or procedures accordingly. |
|  |  |  |  |
