# Supplementary material for: Embedding Technology-Assisted Parenting Interventions in Real-World Settings to Empower Parents of Children With Adverse Childhood Experiences: Co-Design Study
Source: JMIR Form Res. 2024 Nov 22;8:e55639. doi: 10.2196/55639 (PMC11624457; doi:10.2196/55639)
Supplement: Multimedia Appendix 3 [file formative_v8i1e55639_app3.docx]

## Supplementary File 3

**Parent interview schedule**

**Aims:**

1. Evaluate the prototype’s design with parents by understanding if and how the design met parents’ needs, and what if any additional design considerations could better meet parents’ needs.
2. What, if any, additional design considerations could improve meeting these needs [Service provider’s perception of parents’ needs]

**Research Questions:**

- **RQ1:** What was the parents’ experience of listening to the podcasts and engaging with the micro-coaching?
- **RQ2:** In what ways did PaRK-Lite’s design achieve its intended purpose?

**Checklist / script for start of interview**

- Thank participant(s) for attending
- Remind participants of purpose of interview
  - The purpose of this feedback chat is first and foremost to hear your **feedback** on PaRK-Lite we recently completed together.
  - As you know, this program intends to become a part of IPC Health’s services for parents. So I’m also hoping to understand what you think about that too.
  - By understanding this, we’re hoping to make some **final changes to PaRK-Lite** where possible, and come up with a range of strategies that will help IPC Health deliver this program smoothly.
  - It might feel awkward providing feedback that isn’t supportive, but this stage is really about learning about how we can make the program better and easier to use. As such, I would really like you to be **honest with your feedback**, and encourage you to speak as freely as you can - **I will not take any feedback personally**. All feedback is incredibly valuable to the process.
  - As I’ve got recordings of our micro-coaching sessions together, we can spend this chat going over feedback that we haven’t yet talked about- unless there is more you would like to say on what we’ve already spoken about.
- Explain that we have some set questions to ask, but also that we would like to hear any open feedback too.
- Explain that overall interview will take ~60mins, but it’s OK if it’s shorter or longer, we will be guided by how much feedback they have. **Check in with the participant about any time constraints that they may have.**
- Explain that they can change their mind and stop at any time – just let us know.
- Remind about recording. Explain:
  - Recording so that we can transcribe what is said and have an accurate record for our research.
  - The recording will be password protected and stored securely on Monash University servers
  - Only accessible by the research team
  - Stored separately to identifiable information about you, (e.g. your contact details)
  - ASK if they have any questions about this.
- Once confident they understand and consent, explain that you will start recording and then will ask their consent again, for the recording.
- START RECORDING ***Repeat consent questions for recording.*** “Just so I have it on the recording, I’m going to ask again. Based on what I’ve just explained, are you still happy to take part in this interview, including the recording? Great, thank you. Let’s get started…”

| **Construct** | **Question** |
| --- | --- |
| **20mins per topic**  ~6mins per question | **Open-ended opening question:**   - To start with, it would be great to hear your overall impressions of PaRK-Lite’s podcasts and micro-coaching sessions. How would you describe your overall experience with PaRK-Lite?   - What did you like about it? **[PROBE]**   - What did you like the least? **[PROBE]**   - Was anything missing from your point of view? **[PROBE]**   - What do you think could be changed? **[PROBE]**   (Note if any topics below already covered- prioritise others if so) |
| **Technology:**   1. Accessibility 2. Continuity 3. Adaptability | 1. What was your experience with the podcasts like? **[PROBE]**    - Did you encounter any difficulties listening to the podcasts? 2. How likely are you to listen to the podcasts again in the future? **[PROBE:** In what situations?] 3. How tailored did the micro-coaching sessions feel? By tailored, I mean how suited they were to your personal situation and needs. **[PROBE]**    - Tell me about a time where it felt tailored, and/or a time where it did not feel tailored. |
| **Empowerment:**  Reflection   1. (Knowledge acquisition) 2. (Co-constructed knowledge acquisition) 3. (Acting on new knowledge) 4. (Self-efficacy) | 1. What, if any, were some of the biggest take-aways from the podcasts for you? 2. What, if any, were some of the biggest take-aways from the micro-coaching sessions? 3. Have you made any changes to your parenting based on these take-aways, during or after the program?    - If yes, tell me about them, and what you think helped bring about that change.    - If no, why not? 4. How do you feel about doing [topic/strategy] in future? **[PROBE]** |
| **Embeddedness:** | You’ve just completed PaRK-Lite as a stand-alone program that you did in your own time. I’d like you to imagine PaRK-Lite was being offered to you by a service you currently use for you and your child’s health and wellbeing, with a service provider doing the micro-coaching as part of your visit to the service. What are your thoughts on this?  In this scenario, would you be interested in doing PaRK-Lite with a service provider while either waiting for a service, or while receiving another service? **[PROBE:** Why/Why not?]  In this scenario, what (if anything) would help you engage with PaRK-Lite? By engage, I mean choosing to receive PaRK-Lite and sticking with it until you’ve done all the podcasts and micro-coaching. **[PROBE]**   - 1. What kinds of things would make it difficult to engage? |
|  | **Open-ended closing question:**   - Is there anything else you’d like to feedback on PaRK-Lite? Do you have questions or ideas you want to bring up to the team? **[PROBE]** |
| **Conclusion** | - Conclude, summarise key insights - Remind parent that if conversation has brought up anything stressful that they can call helplines (per Information Sheet) - Member checking transcripts- would you like to read over the transcript of our conversation and send back any corrections or clarifications? - Thank for time |

**Elaboration probes**Ensure each response has a ‘because’ to it.

- Can you tell me more about that?

- In what ways?

- How so?

- Why / why is that / why was that the case for you? Why not?

- Why is that important to you?

- Could you give me an example?

_
